# “7/12” patient touch point strategy: a novel method to increase patient attendance and recommendation

**DOI:** 10.1038/s41405-019-0023-y

**Published:** 2019-10-16

**Authors:** Aida Shadrav, Elsbeth Kalenderian, Primitivo Roig

**Affiliations:** 1 Harvard School of Dental Medicine, Boston, MA USA; 2 Department of Preventive and Restorative Dental Sciences, University of California at San Francisco, San Francisco, CA USA; 3 dentalDoctors Institute of Management, Valencia, Spain

**Keywords:** Dental patient management, Preventive dentistry

## Abstract

The US dental system is not likely to see major expansions in dental care use in the foreseeable future. The rise of dental care utilization among low-income children as well as wealthy seniors did not offset flat dental utilization among adults since the Great Recession. Similarly in Spain, the increase in the number of dental practitioners surpasses the rate of population growth. Hence, in order to remain economically viable in this competitive market, one important aspect for every dental office is to retain patients besides attracting new ones. Patients may be lost to follow-up due to a number of factors, including a lack of attention from the dental practice or a lack of trust in the provider. The “7/12" patient touch point strategy of marketing builds consumer loyalty as a result of a strong patient–provider relationship. Furthermore, this method aims to facilitate the patient’s decision to visit their dentist when it is time for preventative treatments. The purpose of this study was to measure the efficacy of a “7/12” patient touch point strategy when compared to the traditional annual recall with respect to number of active patients, percentage of word-of-mouth-recommended patients, and overall number of first-visit patients. We executed the relationship marketing concept through a novel “7/12” patient touch point approach, in which the patient was exposed to seven separate exposures in a period of 12 months. The efficacy of the “7/12” patient touch point was analyzed for 48 months across 10 clinics (24 months before and 24 months after the “7/12” strategy implementation). The “7/12” patient touch point strategy resources consisted of online and printed materials with a focus on oral health knowledge. fter analyzing the efficacy of the “7/12” patient touch point, we found an average increase of 86.91% in the number of active patients, 24.12% in the number of word-of-mouth-recommended patients, and 38.05% in the number of first visits across all clinics. This novel “7/12” patient touch point approach may be successful in increasing the retention of existing patients and generating new patients. Furthermore, this method promotes preventative care and oral health maintanance for patients and economic progress for the dental clinic.

## Introduction

The field of dentistry has seen many changes over the last two decades. For one, practice ownership is declining at a steady rate amongst dentists of all ages.^1^ Second, although the supply of dentists has increased over the years, this has not translated into equal increase of demand of dental services by patients.^2^ Rather, the decline in dental visits during the great recession has not been recovered.^3^ Similarly, in Spain the number of dental professionals will increase by 50% by 2020, while the population growth is expected to increase by merely 2%. This imbalance of supply and demand negatively impacts practice ownership amongst dentists. Trends in the U.S. as well as Spain show that dentists tend to work more often in corporate and group settings.^4^ Therefore, in order to ensure the viability of a dental practice, in addition to the well-being of the patients, novel strategies to ensure patient retention are needed. In general, a strong doctor–patient relationship promotes retention and maximizes patient satisfaction.^5^ In turn, this critical relationship allows for the effective addressing of divergent healthcare beliefs, builds trust, and promotes preventative health care.^6,7^

The American Dental Association^8^ recommends preventative check-ups once or twice a year for examination and regular cleaning.^9^ There is some current thinking that for some patients twice a year might not be needed given their excellent oral health condition and oral health self-care, while others might need more than twice yearly check-up care.^10^ Lack of awareness and education on the importance of oral hygiene is one of the barriers to preventative and self-care.^11^

In advertising, effective frequency is an important theory postulating that a consumer has to be exposed to a product a certain number of times in order to obtain a response.^12^ Although there does not seem to be a specific magic number, general marketing practice recommends the Rule of Seven, stating that the consumer has to be exposed to a product at least seven times in a period of 18 months before it garners their attention.^13^ In addition, ceasing advertisements and connections with the consumer for more than 6 months may irreparably decrease awareness and considerations of the consumer towards the product.^14^ To date, we believe there is no information on the number of educational information a person should receive outside of the dental clinic in order to promote and retain positive oral health self-care habits.

Here, we present on a novel patient education strategy, called the “7/12” patient touch point strategy aiming to connect with the patient seven times in 12 months to promote patient’s awareness of the importance of preventative care and oral health self-care.^1^ These patient touch points include online as well as printed and mailed resources. Patients were emailed newsletters and bulletins emphasizing oral-health and expanding on common oral diseases, e.g., gingivitis and periodontitis. In addition, magazines with educational oral-health topics and annual holiday cards were sent to the patients. This touch point strategy aims to facilitate the patient’s decision to visit their dentist when the time comes for preventative check-ups.

The purpose of this study was to measure the efficacy of the “7/12” patient touch point strategy when compared to traditional annual recall with respect to: number of active patients, percentage of word-of-mouth recommended patients by the active patients, and overall number of first visit completed by the active patients or newly acquired patients through word of mouth.

## Materials and methods

Ten independent clinics, located in Spain, were included in the study within a 48-month period (24 month pre- “7/12” and 24 month post- “7/12” strategy implementation). The clinics on average had 3.0 ± 1.0 chairs, 4.0 ± 1.0 assistants, and 1.5 ± 0.05 office managers. Data was collected by the front desk at the time of patient check-in and stored into the clinic software. The data was categorized into the three following groups:

1. Number of active patients
2. Percent of new patients obtained through word of mouth by the active patients
3. Number of first visits by new patients obtained through word of mouth and walk-in patients

To analyze the effects of the “7/12” patient touch point strategy, descriptive statistics (*mean, standard deviation, minimum, maximum, and median)* was computed and the Wilcoxon test was utilized to compare the data from pre- and post-strategy. Due to the size of the dataset, we have shown the median value along with the mean for a more complete picture. Statistical difference was defined by *P* < 0.05.

The statistical analysis was performed with SPSS, version 15.0 for Microsoft Windows (SPSS Inc., Chicago, IL, USA) by StHalley Statistics.

## Results

To evaluate the effectiveness of the “7/12” patient touch point method with respect to number of active patients, the increases in mean and median were demonstrated for 2-year pre and post-implementation of this strategy (Table 1). The average number of active patients after the implementation of the strategy increased by 86.9%.

**Table 1:** Number of active patients pre and post-7/12 patient touch point strategy

Number of active patients	*N*	Mean	Standard deviation	Minimum	Maximum	Median
**Pre-7/12 method**	10	692.00	437.74	261.00	1644.00	607.50
**Post-7/12 method**	10	1293.40	454.47	591.00	2152.00	1160.00

Furthermore, after implementing the “7/12” patient touch point strategy, the percentage of patients that presented to the clinics recommended by word-of-mouth increased (Table 2). The median has increased by 33.2%, while the mean has increased a total of 24.1%. The last variable, number of first patient visits, shows an increase by 38.05% in the 2 years post-7/12 marketing implementation (Table 3). In the case of first patient visits, there were only eight clinics that were considered (*N* = 8) because there was a digitalization of two clinics that hindered the process of counting first visits, hence why we have put 0% as minimum in the word-of-mouth patients in Table 2.

**Table 2:** Percentage of recommended patients by word of mouth before and after the 7/12 patient touch point strategy

Word of mouth recommended patients	*N*	Mean (%)	Standard deviation (%)	Minimum (%)	Maximum (%)	Median (%)
**Pre-7/12 method**	10	22.55	25.71	0	69.57	15.00
**Post-7/12 method**	10	46.67	20.18	20.00	78.90	48.24

**Table 3:** Number of first patient visits 2 years prior and 2 years post-7/12 patient touch point strategy

Number of first visits	*N*	Mean	Standard deviation	Minimum	Maximum	Median
**Pre-7/12 method**	8	240.13	139.54	115.00	475.00	179.50
**Post-7/12 method**	8	331.50	170.16	160.00	568.00	239.50

The relationship between the “7/12” patient touch point strategy and the number of active patients, percentage of patients acquired by word of mouth, and number of first visits is significant and positive (*p* = 0.005, *p* = 0.005, *p* = 0.012, respectively).

## Discussion

In dentistry, the doctor–patient relationship is crucial for retaining patients. According to the ADA, patients can be categorized into active or inactive patients, where active patients are defined as being seen by the provider within 24 months.^8^ In this study, however, we defined inactive patients as patients who were not seen in the office for 16 months. We used this cut off as a current guidelines’ call to action for at least the annual preventive visit.^15^

The lifetime value of a consumer is defined as the value of cash flow that the consumer may dedicate to a business throughout their relationship.^16^ This can rapidly be translated to the dental setting and as such it is easy to understand that inactive patients have a negative impact on the practice economics. And more importantly, the patient’s oral health, their perception on preventative dental care may also be negatively impacted if they do not see a dentist regularly and/or practice good oral health self-care. Hence, frequent patient education that is patient-focused, cost effective, loyalty driven, and improves retention appears a solid strategy for the modern dental practice.^17^ This proposed methodology appears to support the stated objective.

Building a life-long relationship with the consumer is the foundation of the “relationship marketing” theory.^18^ Relationship marketing focuses on the long-term relationship with the consumer, providing personalized offers while strengthening the network with the consumer for mutual benefits.^19,20^ In 1990, Gronroos further elaborated that “relationship marketing” is a relationship that intends to meet the objective of all parties involved, done by mutual exchange and fulfillment.^21^ Relationship marketing differs from traditional transaction marketing, which focuses on a one-time, single sale with no focus on consumer retention or contact.^22^ When it comes to the practice of healthcare, a strong, longitudinal doctor–patient relationship is imperative, allowing for concepts of relationship marketing to be applied. Healthcare is extremely personal and thus requires extensive effort in order to gain our patients’ loyalty.^23^ Long-term relationships, driven by personalization of care to the patient, will only materialize in a system that understands and caters to the patient’s needs.

Oliver defines loyalty as a deep commitment to rebuy or re-patronize a service despite other situational factors and marketing influences.^24^ Similarly, loyal patients remain with a practice longer, have more preventive check-up visits, and tend to generate more revenues for the practice long term.^25^ Loyalty also saves as much as six times the amount it would take to attract a new patient.^26^ Lastly, loyal patients tend to recommend other patients and those who are recommended to the practice often uphold more allegiance to the practice than patients who present through other services.^27^ Loyalty was not directly assessed in this study, however we believe that we can draw a strong inference. As loyalty is defined as the act of re-patronization of services, we suggest that the 86.9% increase in the number of active patients in this study represents loyalty. Our active patients had one or more visits over the 24-months period of the study. In turn, increased attendance of the patient to their dentist, especially for preventative, periodical check-ups allows the dentist to better assess and manage the patient’s oral health status as well as continuously educate and understand the patient’s needs.

There are many other interventions to promote dental attendance, including sending simple reminders.^28^ Here we focused on “content marketing” and whether sending journals, letters, and blogs to patients can help increase their attendance to their check-up visits and recommendations for their practice. Dentistry, just as all of healthcare, should use ethical marketing to gain the attention of patients about the importance of their oral health status and oral care. These marketing tools include education and providing proactive contents, with the intent that patients will attend their periodic check-up routinely and effortlessly.

## Limitations

Although the statistical data supports the benefits of the “7/12” touch point strategy, there are some study limitations. These include the small number of clinics (*N* = 10), the homogenous make-up of the dental clinics, i.e. all are independent, private clinics owned by dentists, and the fact that all 10- clinics can be considered small to mid-size. As such we cannot make any generalization of our findings. Furthermore, as most pre–post design studies, our study may have been subjected to the Hawthorn effect; however, it provides effective and actionable data at far lower cost and time demands than a randomized controlled trial methodology. We aim to follow the progress of these clinics for a longer duration and look to develop follow up studies to measure specific oral health impact.

## Conclusion

The “7/12” touch point strategy appears to increase attendance and recommendation to the dental clinic.

The “7/12” touch point strategy had a positive impact on the number of active patients, percentage of recommended patients, and number of first visits. This strategy promotest a win–win situation for the patients by promoting oral self-care and preventative check-ups, and enhancing the practice economics for the dental clinic.

## Footnotes

1 Disclosure: The “7/12” patient touch point strategy was developed by dentalDoctors Institute of Management, a private organization focused in training dentists and dental teams in development of new strategies for practice management.
